# Phytochemistry, Antioxidant, and Hepatoprotective Potential of *Acanthospermum hispidum* DC Extracts against Diethylnitrosamine-Induced Hepatotoxicity in Rats

**DOI:** 10.3390/medicines5020042

**Published:** 2018-05-07

**Authors:** Jotham Yhi-pênê N’DO, Adama HILOU, Noufou OUEDRAOGO, Ernest Nogma SOMBIE, Tata Kadiatou TRAORE

**Affiliations:** 1 Laboratory of Biochemistry and Applied Chemistry (LABIOCA), University of Ouaga I Pr Joseph KI-ZERBO, 03 BP 848 Ouagadougou 03, Burkina Faso; hiloudio@gmail.com (A.H.); ernestsombie@yahoo.fr (E.N.S.); 2 Institute for Research in Health Sciences (IRSS/CNRST), Department of Medicine and Traditional Pharmacopoeia (MEPHATRA-PH), 03 BP 7192 Ouagadougou 03, Burkina Faso; ouednouf@gmail.com; 3 Doctoral School of Health, University of Ouaga I Pr Joseph KI-ZERBO, P.O. Box 7021, 03 BP 848 Ouagadougou 03, Burkina Faso; charlkady@gmail.com

**Keywords:** *Acanthospermum hispidum*, diethylenitrosamine, hepatotoxicity, antioxidative enzyme, phenolics, antioxidant activities

## Abstract

**Background:** Burkina Faso is classified among the countries with a high prevalence (˃12%) of hepatitis. Hepatic diseases, such as cirrhosis—related to alcoholism—and hepatitis B and C, are the cause of the increase in cases of liver cancer. They promote the development of cancer by decreasing the natural cell death, causing problems with DNA repair, or by increasing the production of free radical toxins to the cell. According to the World Health Organization (WHO), there were nearly 639,000 deaths from liver cancer worldwide in 2014, hence the need to search for natural hepatoprotective molecules. **Objective:** To evaluate the hepatoprotective potential of *Acanthospermum hispidum* extracts on rats and the antioxidant capacity of extracts in vitro and in vivo, and to perform phytochemistry. **Methods:** The ethanolic and aqueous extracts of the whole *Acanthospermum hispidum* plant were used to evaluate hepatoprotection. The hepatotoxin used in our case was diethylenitrosamine. The animals were divided into groups of six. The sera of the treated animals were used for the determination of transaminases, and the liver homogenates were used for the determination of antioxidant. The total phenol and flavonoid contents, and the antioxidant properties of the extracts, were evaluated in vitro. **Results:** The results of the in vitro antioxidant tests showed good antioxidant activity of the ethanolic extract, using the 2,2-diphenyl-1-picrylhydrazyl (DPPH) test (0.08 ± 0.0018 μg/mL) and 2,2′-azinobis (3-ethylbenzolin-6-sulphonate) (ABTS) (246.05 ± 1.55 mmol TE/g). The in vivo tests showed, through the evaluation of the antioxidant in vivo and the biochemical parameters, that the ethanolic extract with the highest phenolic content had a good hepatoprotective capacity. **Conclusions:** The antioxidant activity of *Acanthopermum hispidum* extracts would justify the observed hepatoprotective activity. These results confirmed that the plant is used in the treatment of liver diseases in traditional medicine in Burkina Faso.

## 1. Introduction

The liver is the target of various attacks (viruses, alcohol, chemical substances present in the diet, or resulting from the living environment) [1]. It also undergoes profound pathological changes, such as in the case of diabetes or obesity [2]. Liver involvement is generally characterized by an increase in certain biochemical parameters, such as transaminases, and a decrease in antioxidant enzymes [3]. Several etiological factors, such as alcohol, hepatitis B or C virus infections, or exposure to aflatoxin-B1, can lead to chronic inflammation and tissue damage, which leads to necrosis of the tissue. Thanks to its quiescent character in a physiological situation, the liver can set up a compensatory proliferation mechanism in a pathological situation [4]. However, long-term cycles of necrosis/regeneration can lead to the appearance of scar tissue (the presence of collagen) and nodules, the latter of which may eventually turn into hepatocellular carcinoma [5]. Interferons are immunomodulatory molecules whose efficacy, after 4–12 months of treatment, is 30–40% in patients that are chronically infected with an HBeAg-positive wild type virus. Its efficacy is also good in cases of HBeAg-negative mutant pre-C virus hepatitis. However, in the latter case, relapses after treatment occur frequently [6]. Over the past twenty years, the incidence of primary liver cancer (hepatocellular carcinoma in 80% of the cases, and cholangiocarcinoma in 10% of the cases) has increased—particularly in developed countries—largely because of an increase in the number of cases of cirrhosis (due largely to the risk factors described above). In Burkina Faso, primary liver cancer is the leading cause of out-of-country health evacuation and the third leading cause of death, after infectious diseases and cardiovascular diseases [7]. At present, the detection methods are too sensitive to consider an early diagnosis. Additionally, accuracy and the lack of therapeutic alternatives for the management of patients with hepatocarcinoma, make liver cancer a very poor prognosis. According to Professor Alain Bougouma [8], the treatment of hepatitis could cost 909 U.S. dollars per month per patient. Hepatitis puts a heavy burden on the health care system, because of the difficulties associated with the medical management of these complications, as well as the cost of conventional drugs. Faced with the limitation of therapeutic alternatives, the identification of new molecules—especially of natural origin—represents an important issue.

*Acanthospermum hispidum* DC (Asteraceae) is a herb that was selected from an ethnobotanical survey in 2014 that identified the medicinal plants that were used in the management of liver diseases by traditional medicine in Burkina Faso [9]. The presented work consisted of evaluating the hepatoprotective properties of this plant, and then performing a phytochemical investigation—with in vitro antioxidant capacity—of the two forms of extract, in order to respect the methods of use proposed by the traditional health practitioner.

## 2. Materials and Methods 

### 2.1. Plant

The plant material consists of the whole *Acanthospermum hispidum* DC plant, which was harvested in 2016 in Loumbila. The plant was identified at the Laboratory of Plant Ecology and Botany of the University Ouaga I Pr Joseph KI-ZERBO. The specimens were deposited at the herbarium of the Biodiversity Laboratory under identification code ID 16875. The plants were dried with MEPHATRA-Ph at room temperature and out of the sun, and then the dry plant material was returned in powder form.

### 2.2. Chemical Equipment

Sigma reagents (Steinheim, Germany): sodium phosphate monobasic (NaH_2_PO_4_), sodium phosphate dibasic (Na_2_HPO_4_), ethylenediamine tetraacetic acid (EDTA), 2,2-diphenyl-1-picrylhydrazyl (DPPH), 2,2′-azinobis (3-ethylbenzolin-6-sulphonate) (ABTS), 2-2-deoxy d-ribose, hydrogen peroxide, nitro chloride blue tetrazolium, sylimarine, diethylnitrosamine (DEN), ascorbic acid, gallic acid, quercetin, trolox.

Fluka chemie reagents (Buchs, Switzerland) and prochimie: potassium hexacyanoferrate [K_3_Fe (CN)_6_], iron chloride [FeCl_3_], iron sulphate, trichloroacetic acid and thiobarbituric acid. They were all of analytical grade.

### 2.3. Extraction

#### 2.3.1. Ethanol Maceration

The whole plant powder of *Acanthospermum hispidum* was macerated (at about 30 °C) in absolute ethanol at a ratio of 1:5 (mass/volume) for 24 h with mechanical stirring. The macerated whole plant powder was filtered. The filtrate that was obtained (brown-green, oily in appearance) was concentrated in an evaporator that was equipped with a vacuum pump. The extract that was obtained was frozen at minus 20 °C and freeze-dried for subsequent investigations.

#### 2.3.2. Aqueous Decoction

It was weighed precisely in a test portion of 100 g of plant material in a 1000 mL ground-necked flask, to which 500 mL of distilled water was added. The mixture was homogenized and boiled under reflux for 30 min, after which it was allowed to warm up. The contents of the flask were spilled in centrifuge tubes. Finally, the supernatant was concentrated, frozen and freeze-dried.

### 2.4. Phytochemical Investigation

#### 2.4.1. Determination of Total Polyphenols

The determination of total phenolic was carried out according to the method described by Compaoré et al. [10], with some modifications. This method is based on colorimetry, using Folin ciocalteu. One hundred and twenty-five microliters of Folin reagent (0.2 mol/L) was added to 25 μL of extract. The reaction mixture was then allowed to incubate for 2 h at room temperature. A spectrophotometer reading, using a microplate reader, was taken at 760 nm. Gallic acid was used as a reference to plot the standard curve (y = 201x − 21.22, *r*^2^ = 0.99) and the result was expressed in milligram Equivalent Gallic acid per gram of extract (mg GAE/g).

#### 2.4.2. Determination of Total Flavonoids

The flavonoid assay was performed according to the method described by Compaoré et al. [10]. One hundred microliters of AlCl_3_ (2%) was added to the extract (1 mg/mL). The mixture was made in the microplate wells and then allowed to incubate for 15 min. The reading was taken at 415 nm against a quercetin standard curve (y = 39.8x − 3.5, *r*^2^ = 0.99) and the amounts were expressed in milligram Equivalent Quercetin per gram of extract (mg QE/g).

### 2.5. Determination of the Antioxidant Potential of the Ethanolic and Aqueous Extract of Acanthospermum hispidum

In order to evaluate—in vitro—the antioxidant potential of *Acanthospermum hispidum* extracts, antioxidant models (DPPH, ABTS, and FRAP) were used. Several factors intervene in the measurement of the antioxidant capacity of a matrix, namely, the chemical reactivity of the antioxidant with respect to the radical that is to be trapped, the destination of the radical that is derived from the antioxidant, the interaction with other antioxidants, concentration and surrounding mobility, absorption, distribution, retention, and metabolism of the antioxidant.

#### 2.5.1. Inhibition of Radical ABTS Assay

This method was based on the decolorization of the stable radical cation ABTS^•+^ [2,2′-azinobis-(3-ethylbenzothiazoline-6-sulfonic acid)]. The disappearance of the blue-green chromophoric radical ABTS^•+^ was monitored at 734 nm, according to the method described by Re et al. [11]. The sample that was to be tested (500 μg/mL in 50 μL of methanol) was incubated for 15 min in the dark with 200 μL of freshly prepared ABTS^•+^ solution. The absorbance at 734 nm was measured, using a spectrophotometer, against a standard trolox curve (y = −72.38x + 54.57, *r*^2^ ˂ 0.99, *p* ˂ 0.001). The experiment was carried out in triplicate (independent tests) and the anti-radical activity of the extract, by reduction of the radical cation ABTS^•+^, was expressed in millimoles Equivalent Trolox per gram of extract (mmol TE/g).

#### 2.5.2. Reduction of Iron III Assay (FRAP)

The reducing power of the extracts method was based on the reduction of ferric ion (Fe^3+^) to ferrous ion (Fe^2+^) by the reducing compounds, following a mono-electron transfer. The determination of the reducing power of *Acanthospermum hispidum* extracts was evaluated as described by Hinneburg et al. [12], with some modifications. In a test tube, 1.25 mL of phosphate buffer (0.2 M, pH 6.6) and 1.25 mL of potassium hexacyanoferrate (1% aqueous) were added to 0.5 mL of test extract (0.6 mg/mL). The mixture was heated at 50 °C in a water bath for 30 min. After cooling, trichloroacetic acid (1.25 mL, 10%) was added and then the mixture was centrifuged (2000× *g* for 10 min). Three aliquots (125 μL) of the supernatant were transferred into a 96-well microplate, after which 125 μL of distilled water and then 25 μL of FeCl_3_ (0.1% aqueous) were added. The reductive power was evaluated at 700 nm, against a standard curve of ascorbic acid (y = 105.9x, *r*^2^ > 0.99, *p* < 0.0001). The experiment was carried out in triplicate (independent tests) and the reducing activity of the extract was expressed in milligram Equivalent Ascorbic Acid per gram of extract (mg AAE/g).

#### 2.5.3. Inhibition of Radical DPPH Assay

The anti-radical activity of the crude extracts was evaluated by the DPPH (2,2-diphenyl-1-picrylhydrazyl) method, as described by Alisi et al. [13]. This method is based on the reduction of the absorbance of the stable free radical DPPH^•^ at 517 nm, in the presence of an H^•^ radical donor. A series of seven successive dilutions (at 1/2) was carried out from a stock solution (10 mg/mL in methanol) of ethanolic and aqueous extracts of *Acanthospermum hispidum*. The test sample (100 μL in methanol) was mixed with 200 μL of DPPH and then incubated at room temperature for 15 min. Absorbance was measured at 517 nm against a blank, using a spectrophotometer. The evaluation of the anti-radical properties of the sample made it possible to calculate the percentages of reduction of the radical DPPH^•^ by decreasing the concentrations of the extracts. The experiment was carried out in triplicate (independent tests) and the concentrations, (μg/mL) trapping 50% of free radicals (IC50), were determined using the curves of the percentages of anti-radical activities as a function of the concentrations of the extracts.

### 2.6. Biomembrane Protection

#### 2.6.1. Desoxyribose Degradation Inhibitory Assay

The hydroxyl radical is a far more reactive secondary free radical than its precursors (superoxide anion, hydrogen peroxide). The ability of the extracts to trap the hydroxyl radical was evaluated using the desoxyribose degradation scavenging assay, as described by Perjési and Rozmer [14]. The hydroxyl radical is produced in situ by iron sulfate, which decomposes hydrogen peroxide into a hydroxyl radical. The hydroxyl radical that is formed attacks the desoxyribose (a component of the DNA) at the C_2′_ level and causes the opening of the ring with the formation of malonylaldehyde. This product, with thiobarbituric acid, forms a pink complex that is dosed at 532 nm. The reaction mixture consisted of 100 μL of the extract (1 mg/mL in a 50 mM phosphate buffer, pH 7.4), 100 μL of EDTA (1.04 mM aqueous), 100 μL of iron sulfate (100 μL aqueous mM), 100 μL of desoxyribose (60 mM aqueous), and 100 μL of hydrogen peroxide (10 mM). The volume was increased to 1 mL with a phosphate buffer, and then the mixture was incubated (37 °C for 1 h). Trichloroacetic acid (1 mL, 15% aqueous) and thiobarbituric acid (1 mL, 0.675% in 25 mM aqueous NaOH) were added and the whole mixture was then incubated (100 °C for 15 min). After cooling in an ice bath (5 min), the tubes were centrifuged (3000× *g* for 10 min) and then 200 µL of the supernatant was transferred into 96-well microplates. The trapping of desoxyribose degradation was measured, using a spectrophotometer, at 532 nm against a blank. Gallic acid was used as a reference substance. The experiment was carried out in triplicate (independent tests) and the ability of the extract to trap desoxyribose degradation was expressed as a percentage of desoxyribose degradation trapping, which was compared to a control without the extract.

#### 2.6.2. Lipid Peroxidation Inhibitory Assay

It is also important to evaluate the ability of a matrix to inhibit the initiation and the spreading of lipid peroxidation. The ability of *Acanthopermum hispidum* extracts to inhibit lipid peroxidation was evaluated using lecithin liposomes as a membrane model, according to the method described by Su et al. [15]. The reaction medium consisted of 100 μL of the extract (1 mg/mL in a 10 mM phosphate buffer, pH 7.4), 100 μL of iron sulfate (100 mM), 100 μL of EDTA (1.04 mM), 100 μL of hydrogen peroxide (10 mM), and 100 μL of an opalescent suspension of lecithin (10 mg/mL in a phosphate buffer). The reaction volume was increased to 1 mL with a phosphate buffer and then the mixture was incubated (37 °C for 1 h). The reaction was halted by adding 1 mL of 0.25 N hydrochloric acid, containing 15% trichloroacetic acid and 0.675% thiobarbituric acid. The mixture was then heated to 100 °C for 15 min and cooled in an ice bath for 5 min. After centrifugation (3000× *g* for 10 min), the absorbance of the supernatant was measured, using a spectrophotometer, at 532 nm against a blank. Gallic acid was used as a reference substance. The experiment was carried out in triplicate (independent tests) and the inhibition of lipid peroxidation by the extract was expressed as the concentrations (μg/mL) inhibiting the peroxidation of 50% of lipids (IC50). The IC50 values were determined by the percentage of inhibition curves as a function of the extract concentrations.

### 2.7. In Vivo Hepatoprotection

#### 2.7.1. Animal Conditioning

The experimental animals were male rats of Wistar variety, weighing between 220–250 g. All animals were FSPO (free of specific pathogenic organisms) sanitary status.

Upon receipt, the rats were randomly placed in groups of six in standard cages for an acclimation period (2 weeks), before being used in the various experiments. During this period, the animals had free access to food and water (kibble from the animal feed production company) and were kept in a constant temperature (22 ± 2) °C pet shop, subject to a light/dark cycle of 12/12 h. The dark phase of this cycle began at 12 p.m. and the different experiments always took place from 1 p.m. to 6 p.m., due to the nocturnal activity of the animal (active phase). All experimental animal protocols had complied with the instructions of the Institutional Animal Ethics Committee (directive 2010/63/EU on protection of animals used for scientific purposes). Ethical approval code: 2010/63/EU, Date of approval: 20 October 2010.

#### 2.7.2. Animal Treatment

The animals were distributed as follows:

Lot I (normal) received distilled water by gavage; Lot II (control) received distilled water orally; Lot III received sylimarine, a reference antioxidant, at the daily dose of 50 mg/kg of oral body weight [16]. The test lots (IV, V, VI, VII, VIII, and IX) received a variable dose of the test extracts once a day (50, 100, 250 mg/kg of body weight). All of the treatments were administered orally. On day 7, with the exception of lot I, animals received intraperitoneally diethylnitrosamine (DEN) (200 mg/kg body weight). On the eighth day, all rats were sacrificed.

#### 2.7.3. Biochemical Parameters

Blood was drawn by puncturing the retro-orbital plexus under diethyl ether anesthesia. Whole blood, for hematogram, was collected in bottles that contained the anticoagulant, ethylene diamine tetraacetic acid (EDTA), while samples for biochemical analysis were collected in plain sample bottles. Serum was separated by centrifugation at 3000× *g* for 15 min and analyzed for various biochemical parameters. Alanine aminotransferase (ALAT) and aspartate aminotransferase (ASAT) were assayed using a kit (LABKIT) according to the manufacturer’s instructions.

#### 2.7.4. Assay of Antioxidant Enzymes and Malondialdehyde In Vivo

Liver tissues were homogenized in a 0.1 M tris buffer (pH 7.0) and centrifuged at 12,000× *g* for 10 min. The supernatant was used for the measurement of liver enzymatic and non-enzymatic antioxidants. Therefore, the antioxidant activity of the extracts was evaluated both by measuring the level of oxidation of lipids, and by measuring the activity of the enzymes involved in the body’s defense against the phenomena of oxidation. The level of malondialdehyde in the liver was measured according to the method set out by Ohkawa et al. [17]; Superoxide dismutase was tested using the standard method revealed by Misra and Fridovich [18]; Catalase was measured using a standard protocol given by Beers and Siezer [19].

#### 2.7.5. Statistical Evaluation

In the tables, the data were expressed in Mean ± SD. The graphs were drawn, and the statistical analysis was carried out, using GraphPad Prism soſtware version 5.0 for Mac OS X (GraphPad Soſtware, San Diego, CA, USA).

## 3. Results

### 3.1. Antioxidant Activities

The antioxidant capacity of the extracts was evaluated according to three methods (Table 1). Firstly, with respect to the ability of the extracts to trap the DPPH radical, a better concentration of 50% inhibition (Ic 50%) of the DPPH radicals with the ethanolic extract (0.08 ± 0.0018 μg/mL) and a relatively low inhibition concentration with the aqueous extract was noted. Secondly, the ABTS radical anion reduction power that was evaluated showed that the ethanolic extract was the most active (246.05 ± 1.55 mmol TE/g) when compared with the aqueous extract (57.325 ± 1.26 mmol TE/g). Finally, the ion reduction capacity of the crude extracts that were determined showed that the aqueous extract had a good reduction (856.14 ± 2.59 mg AAE/g) relative to that of the ethanolic extract (336.05 ± 2.91 mg AAE/g).

### 3.2. Biomembrane Protection

The protective power of the extracts’ biomembranes was evaluated with respect to their ability to inhibit the peroxidation of membrane lipids and the degradation of d-desoxyribose (Table 2). Initially, the inhibition of lipid peroxidation, with the ethanolic extract of *Acanthospermum hispidum*, had given a peroxidation inhibition concentration of 50% of lipids that was relatively good (40 ± 1.3 μg/mL) compared with the aqueous extract (181 ± 4.5 μg/mL) and the gallic acid (reference compound). In the second step, concerning the inhibition of the degradation of d-desoxyribose, it was found that all of the extracts showed good activity for an initial concentration of 1 mg/mL. The best percentage of inhibition of the degradation was recorded in the crude ethanolic extract (90.86 ± 1.52%).

### 3.3. Phytochemistry

Determination of total phenolic and total flavonoids

The total phenolic assay showed that the ethanolic crude extract of *Acanthospermum hispidum* is richer (335.8 ± 6.30 mg EAG/g) than the aqueous extract (312.4 ± 5.6 EAG/g) (Table 3). The total flavonoid assay confirmed that the extracts contain as many flavonoids as other types of phenolic compounds. The ethanolic extract had the highest content of total flavonoids (24.17 ± 6.95 mg EQ/g extract). The number of flavonoids varied from 19.85 ± 9.65 to 24.17 ± 6.95 mg EQ/g.

### 3.4. Hepatoprotective Activity In Vivo

#### 3.4.1. Antioxidant Enzymes and Malondialdehyde In Vivo

##### Variation of Catalase (CAT)

**Figure 1:**
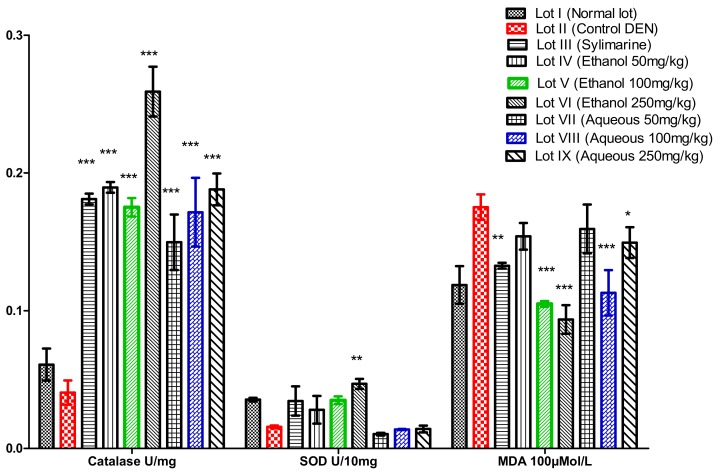
Variation of catalase, superoxide dismutase and malondialdehyde; *p* > 0.05: the difference is not significant; 0.05 > *p* > 0.01: the difference is significant *; 0.05 > *p* > 0.001: the difference is highly significant **; *p* < 0.001: the difference is very highly significant ***. Compared with the positive control (diethylnitrosamine (DEN)).

The results of the catalase assay for rat liver homogenates showed that, compared with the control and control negative rats, there was a statistically significant increase (*p* < 0.01) in the amount of catalase animals treated with extracts (Figure 1). The highest dose of catalase was recorded in the liver homogenates of the rats that were treated with the ethanolic extract at 250 mg/kg body weight, with a value of 0.26 ± 0.051 U/mg protein. The variation in the number of catalases represents a significant difference (*p* < 0.05) between the catalase variation of the animals treated with the aqueous extract (0.18 ± 0.0007 U/mg) and that of the animals treated with the ethanolic extract (0.207 ± 0.012 U/mg).

##### Variation of Superoxide Dismutase (SOD)

The treatment of the rats with the ethanolic and aqueous extract of *Acanthospermum hispidum* at 50, 100, and 250 mg/kg body weight led to a significant increase in SOD enzyme activity of the liver homogenates (Figure 1). Thus, for the liver homogenate, high activity was observed in the rats that were treated with the extract at the dose of 250 mg/kg body weight, with 0.0469 ± 0.0007 U/10 mg of protein. However, the activity was impaired in negative control rats (DEN) with 0.016 ± 0.001 U/10 mg of protein. The variation of superoxide dismutase between the negative treated rats and the negative control rats was significant (*p* ≥ 0.001). On the other hand, the variation between the rats that were treated with the ethanolic extract and those that were treated with the aqueous extract was not significant (*p* > 0.05).

##### Variation of Malondialdehyde (MDA)

Compared with rats in the negative control lot, the rats that were treated with three doses of extract showed a significant decrease in MDA levels. The largest decrease was recorded in the rats that received the *Acanthospermum hispidum* extract at a dose of 250 mg/kg body weight, with 0.094 ± 0.0008 × 100 μM/L (Figure 1). A high peroxidation of fatty acids was observed in toxin treated rats (DEN), with a significant increase (*p* < 0.001) with 0.135 ± 0.021 × 100 μM/L. An analysis of the rat liver fatty acid peroxidation assay results showed that there was an increase in the level of MDA in the negative control rats (*p* < 0.001) compared with rats treated with the *Acanthospermum hispidum* extracts (100 mg/kg body weight for the aqueous extract and 250 mg/kg body weight for the ethanolic extract). Similarly, a significant difference was observed between the amount of MDA in the rats treated with the ethanolic extract (0.0702 ± 0.002 × 100 μM/L) and those treated with the aqueous extract (0.113 ± 0.01 × 100 μM/L) at a dose of 100 mg/kg body weight.

#### 3.4.2. Biochemical Parameters

##### Assay for Alanine Aminotransferase (ALAT) and Aspartate Aminotransferase (ASAT)

**Figure 2:**
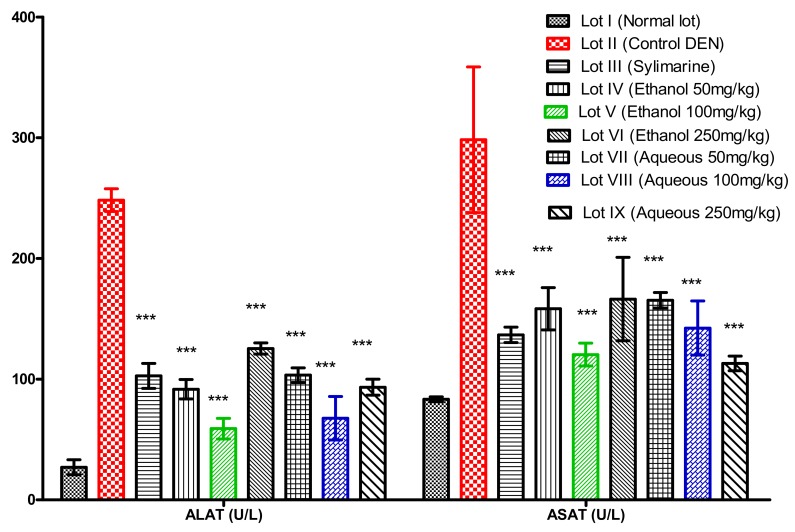
Assay results of alanine aminotransferase (ALAT) and aspartate aminotransferase (ASAT); *p* < 0.001: the difference is very highly significant ***. Compared with the positive control (DEN).

The different lots that were made for this purpose had undergone the appropriate treatments. Twenty-four hours later, the animals were sacrificed and the blood samples that were collected were used for the determination of these transaminases. The results that were obtained were expressed in International Units per liter (U/L) and were represented in the form of the means plus or minus the standard deviations for the different batches. ALAT and ASAT are enzymes that play a role in the metabolism of pyruvic and oxaloacetic acids. The values of the transaminases (Figure 2) showed that there was a significant difference between the values of the animals treated with the extracts and those of the negative control (*p* < 0.001). When compared with animals in the normal batch, the ASAT and ALAT values of the animals treated with the ethanolic and the aqueous extract at 100 mg/kg body weight showed that there was no significant difference, with 120.33 ± 8.3 U/L and 59 ± 4.2 U/L; 142.33 ± 11.8 U/L and 67.66 ± 6.8 U/L. With respect to sylimarine, it was found that for ASAT, there was no significant difference between the animals treated with the extracts and those treated with sylimarine (reference compound). The dose of 100 mg/kg of the ethanolic extract showed a better number of transaminases relative to the animals in the positive control lot (treated with sylimarine). The batches that were treated with the *Acanthospermum hispidum* extracts had lower ASAT and ALAT levels than the negative control lot (Figure 2). They also showed a statistically significant difference (*p* < 0.01) between the level of ALAT in the animals of the lots where the ethanolic extract and the aqueous extract of *Acanthospermum hispidum* (100 mg/kg) were administered in a preventive mode, and of those where the animals received sylimarine (reference compound).

## 4. Discussion

Following the analysis of the results of the antioxidant tests, we found that all of the extracts showed antioxidant activity. The ethanolic extract of *Acanthospermum hispidum* produced an interesting DPPH radical inhibition capacity relative to quercetin, with 0.044 μg/mL (reference compound). These results were different from those of some of the other authors, such as Mothana et al. [20]. This difference could be explained by the difference in the parts that were used and by many other factors, such as edaphic factors for instance. The antioxidant capacity of the extracts could justify their hepatoprotective potential [21]. The presence of phenols and flavonoids, which are recognized for their high antioxidant capacity, in the extracts justifies the observed antioxidant power [22].

Biological membranes, such as hepatocytes membranes, consist of lipids and complex carbohydrates [23]. The radicals that form initiate the lipid peroxidation of the biomembrane by an electrophilic attack that propagates through the generated alkoxyl and peroxyl radicals [24]. The consequence of this is the degradation of the integrity of the membrane. The extracts that have been shown to inhibit lipid peroxidation and desoxyribose degradation could either directly eliminate the hydrogen peroxide by transforming it into a water molecule [4], or exert a positive action on the molecule. The transmembrane ionic motion is necessary to maintain the integrity of the hepatocyte membrane. Considering the antioxidant potential highlighted in this study, the extracts of *Acanthospermum hispidum* would protect biomembranes by directly trapping the hydroxyl radicals that are preventing the initiation and propagation of membrane lipid peroxidation, and the membrane cell carbohydrates of liver cells [4]. The polyphenolic compounds of the extracts could further induce an increase in membrane fluidity, reducing the interaction of oxidants with membrane phospholipids. These properties are attributed to phenolic compounds [25].

Among the cellular enzymatic antioxidant systems are the superoxide dismutase and catalase [24]. The superoxide anion, the first toxic species formed from oxygen, is removed and maintained by the SOD at a low level of concentration, which catalyzes its dismutation in H_2_O_2_. The latter is converted into H_2_O and O_2_ by the catalase [26]. A biochemical evaluation of the activity of the antioxidant enzymatic systems (catalase and superoxide dismutase) revealed that there was a significant reduction in the cytosolic activity of these enzymes in the liver of the animals that were treated with DEN. At the same time, pretreatment of the rats with the *Acanthospermum hispidum* extracts was able to maintain the antioxidant cellular defense systems (CAT and SOD) at their normal cell level, thereby preventing the loss of the antioxidant/redox balance that is found in the animals that were not protected by the extracts of *Acanthospermum hispidum*.

In animals treated with DEN only, the catalase levels were significantly reduced by DEN free radical production action [27]. This phenomenon also causes depletion and facilitates the lipid peroxidation and the oxidation of the thiol groups of the proteins [28]. However, pretreatment of the animals with the *Acanthospermum hispidum* extracts prevented the decrease of catalases and SOD observed in the rats that were receiving hepatotoxin alone. The extracts of *Acanthospermum hispidum* capture the free radicals produced by DEN [29] by their phenolic compounds. Palozi et al. [30] also reported a good antioxidant capacity of extracts of the aerial part of *Acanthospermum hispidum* in vivo.

DEN poisoning is defined as hepatic cytolysis syndrome [28]. This toxic attack results in a significant increase in the biochemical markers, which are characteristic of this syndrome (serum transaminases), compared to normal [31]. In this case, the significant increase in the serum transaminase levels (mainly ALAT) of the negative control lot compared with the normal control group, and those treated with the extracts (*p* < 0.001), clearly indicates that the intoxication is effective and exercises mainly on the liver. They are found in the blood in the case of lesion or membrane rupture (alteration or destruction of cells, including cytolysis during necrosis) [32,33]. The administration of plant extracts in preventive mode resulted in a significant inhibition of the increase in transaminase levels. This may make it possible to assert the existence of an anti-hepatotoxic effect of the *Acanthospermum hispidum* extracts at the doses that were used. However, the decreasing transaminase levels observed for the lots treated with the ethanolic extract should be taken into account, because the chemical composition of the ethanolic extract favors the use of *Acanthospermum hispidum* as a hepatoprotective plant.

## 5. Conclusions 

The results of the assays showed that the ethanolic extract is as rich in total phenolic and total flavonoids as the aqueous extract. The phenolic compounds in the ethanol extract were confirmed by the high levels of the antioxidant activities, evaluated in vitro by the ABTS and DPPH tests, and in vivo by the catalase, superoxide dismutase, and malondialdehyde assay. The antioxidant activity, combined with the antihepatotoxic activity of the *Acanthospermum hispidum* extracts, could justify the use of the plant in the treatment of liver diseases in traditional medicine in Burkina Faso.

## Figures and Tables

**Table 1:** Results of antioxidant tests.

Extract	Antioxydant Activities		
	DPPH Ic 50% (µg/mL)	ABTS (mmol TE/g)	FRAP (mg AAE/g)
Ethanol extract	0.08 ± 0.0018 ^b^	246.05 ± 1.55 ^a^	336.05 ± 2.391 ^b^
Aqueous extract	0.6 ± 0.0012 ^c^	57.325 ± 1.26 ^b^	856.14 ± 2.59 ^a^
Quercetin	0.044 ± 0.008 ^a^	-	-

Each value represents mean ± SEM; the difference is significant (*p* > 0.05) for letters (a to c).

**Table 2:** Results of inhibition of lipid peroxidation and degradation of deoxyribose.

Extract	Protection of the Biomembranes	
	Lipid Peroxidation Extract Ic 50% (μg/mL)	Degradation of Desoxyribose Inhibition (% Inhibition)
Ethanol extract	40 ± 1.3 ^a^	90.86 ± 1.52 ^a^
Aqueous extract	181 ± 4.5 ^c^	75.87 ± 2.12 ^c^
Gallic Acid (reference)	42 ± 1.8 ^b^	84.68 ± 3.31 ^b^

Each value represents mean ± SEM; the difference is significant (*p* > 0.05) for letters (a to c).

**Table 3:** Results of total phenolics and total flavonoids.

Extract	Phytochemistry	
	Total Phenolic (mg EAG/g Extract)	Total Flavonoids (mg EQ/g Extract)
Ethanol extract	335.8 ± 6.30 ^a^	24.17 ± 6.95 ^a^
Aqueous extract	312.4 ± 5.6 ^b^	19.85 ± 9.65 ^b^

Each value represents mean ± SEM; the difference is significant (*p* > 0.05) for letters (a to b).

## References

[1] Parsa N. (2012). Environmental factors inducing human cancers. Iran. J. Public Health.

[2] Cowart L.A. (2009). Sphingolipids: Players in the pathology of metabolic disease. Trends Endocrinol. Metab..

[3] Michel F., Mas E., Drai J. (2008). Biomarqueurs de la peroxydation lipidique: Aspects analytiques. Ann. Biol. Clin..

[4] Sarita N., Uyanik F., Hamurcu Z., Çoksevim B. (2011). Effects of acute twelve minute run test on oxidative stress and antioxidant enzyme activities. Afr. J. Pharm. Pharmacol..

[5] Ibrahim A.S., Zaghloul H., Badria F.A. (2013). Case Report Evidence of Relationships between Hepatocellular Carcinoma and Ochratoxicosis. PLoS ONE.

[6] Chang T.T., Gish R.G., Hadziyannis S.J., Cianciara J., Rizzetto M., Schiff E.R., Pastore G., Bacon B.R., Poynard T., Joshi S. (2005). A dose-ranging study of the efficacy and tolerability of entecavir in lamivudine-refractory chronic hepatitis B patients. Gastroenterology.

[7] (2013). Plan Stratégique de Lutte Contre Le Cancer 2013–2017.

[8] Birama D. (2017). Epidémiologie Moléculaire du Virus de L’hépatite B au Burkina Faso: Séroprévalence, r ôle de l’APOBEC3G d ans la Coïnfection VHB/VIH-1, Détermination des cas D’infection Occulte, Séquençage et Caractérisation des Génotypes.

[9] (2014). FONRID, Les Projets Issus Du 3. 2014.

[10] Compaoré M., Lamien-Meda A., Mogoşan C., Lamien C.E., Kiendrebeogo M., Voştinaru O., Vlase L., Ionescu C., Nacoulma O.G. (2011). Antioxidant, diuretic activities and polyphenol content of *Stereospermum kunthianum* Cham. (Bignoniaceae). Nat. Prod. Res..

[11] Re R., Pellegrini N., Proteggente A., Pannala A., Yang M., Rice-Evans C. (1999). Antioxidant activity applying an improved ABTS radical cation decolorization assay. Free Radic. Biol. Med..

[12] Hinneburg I., Dorman D., Hiltunen R. (2006). Antioxidant activities of extracts from selected culinary herbs and spices. Food Chem..

[13] Alisi C.S., Ojiako O.A., Osuagwu C.G., Onyeze G.O.C. (2011). Free Radical Scavenging and In-vitro Antioxidant Effects of Ethanol Extract of the Medicinal Herb *Chromolaena odorata* Linn. Br. J. Pharm. Res..

[14] Perjési P., Rozmer Z. (2011). Kinetic analysis of some chalcones and synthetic chalcone analogues on the fenton-reaction initiated deoxyribose degradation assay. Open Med. Chem. J..

[15] Su X.-Y., Wang Z.-Y., Liu J.-R. (2009). In vitro and in vivo antioxidant activity of *Pinus koraiensis* seed extract containing phenolic compounds. Food Chem..

[16] Reznik G.K., Padberg G. (2000). Diethylnitrosamine-induced metastasizing hepatocellular carcinomas in New Zealand white rabbits A tumor model for clinical investigations. J. Cancer Res. Clin. Oncol..

[17] Ohkawa H., Ohishi N., Yagi K. (1979). Assay for lipid peroxidation in animal tissues by thiobarbituric acid reaction. Ann. Biochem..

[18] Misra H.P., Fridovich I. (1972). The Role of Superoxide Anion in the Epinephrine and a Simple Assay for Superoxide Dismutase Autoxidation of. J. Biol. Chem..

[19] Beers F., Sizer J.R. (1951). Cambridge, a spectrophotometric method for measuring the breakdown of hydrogen peroxide by catalase. J. Biol. Chem..

[20] Mothana R.A., Lindequist U., Gruenert R., Bednarski P.J. (2009). Studies of the in vitro anticancer, antimicrobial and antioxidant potentials of selected Yemeni medicinal plants from the island Soqotra. BMC Complement. Altern. Med..

[21] Fardet A. (2017). Le pouvoir antioxydant des produits laitiers une propriété méconnue de leur potentiel protecteur. Choledoc.

[22] Achat S. (2014). Polyphénols de l’alimentation: Extraction, Pouvoir Antioxydant et Interactions avec des ions Métalliques.

[23] Futerman A.H., Hannun Y.A. (2004). The complex life of simple sphingolipids. EMBO Rep..

[24] Mohamed K. (2009). Activité Biochimique des Extraits Flavonoïdiques de la Plante Ranunculus Repens L: Effet sur le Diabète Expérimental et L’hépatotoxicité Induite par l’Epirubicine. Ph.D. Thesis.

[25] Methorst C., Huyghe E. (2014). Volume 24—Septembre 2014—Hors-série 3 Stress oxydant et infertilité masculine: Physiopathologie et intérêt thérapeutique des antioxydants et les membres du Comité d’Andrologie et de Médecine Sexuelle de l’Association Française d’Urologie Sous-Comité Fe. Progrès en Urol..

[26] Popovici C., Saykova I., Tylkowski B. (2009). Evaluation de l’activité antioxydante des composés phénoliques par la réactivité avec le radical DPPH. Rev. Génie Ind..

[27] Benkhedir A., Madihadjoue M. (2016). Contribution à L’Etude De L’Effet Du Thymus Numidicus Sur L’Hepatotoxicite Induite par L’Alloxane chez La Souris.

[28] Fusco D., Colloca G., Lo-Monaco M.R., Cesari M. (2007). Effects of antioxidant supplementation on the aging process. Clin. Interv. Aging.

[29] Woreta T., Alqahtani S. (2014). Evaluation of abnormal liver tests. Med. Clin. N. Am..

[30] Palozi R.A.C., Schaedler M.I., Tirloni C.A.S., Silva A.O., Lívero F.A.D.R., Souza R.I.C., dos Santos A.C., Prando T.B.L., de Souza L.M., Gasparotto Junior A. (2017). Roles of Nitric Oxide and Prostaglandins in the Sustained Antihypertensive Effects of *Acanthospermum hispidum* DC. on Ovariectomized Rats with Renovascular Hypertension. Evid.-Based Complement. Altern. Med..

[31] Thapa B.R., Walia A. (2007). Liver Function Tests and their Interpretation. Indian J. Paediatr..

[32] Bergsbaken T., Fink S.L., Cookson B.T. (2010). Pyroptosis: Host cell death and inflammation. Nat. Rev. Microbiol..

[33] Guicciardi M.E., Malhi H., Mott J.L., Gores G.J. (2013). Apoptosis and Necrosis in the Liver Maria. Compr. Physiol..

